# Behavioral training rescues motor deficits in *Cyfip1* haploinsufficiency mouse model of autism spectrum disorders

**DOI:** 10.1038/s41398-018-0338-9

**Published:** 2019-01-21

**Authors:** Sven O. Bachmann, Monika Sledziowska, Ellen Cross, Shireene Kalbassi, Sophie Waldron, Fangli Chen, Adam Ranson, Stéphane J. Baudouin

**Affiliations:** 1 School of Biosciences, Cardiff University, Cardiff, CF10 3AX Wales UK; 2 Neuroscience and Mental Health Research Institute, School of Medicine, Cardiff University, Cardiff, CF24 4HQ UK; 3 Faculty of Medicine and Health Sciences, Universitat Internacional de Catalunya, Barcelona, Spain

## Abstract

Deletions in the 15q11.2 region of the human genome are associated with neurobehavioral deficits, and motor development delay, as well as in some cases, symptoms of autism or schizophrenia. The cytoplasmic FMRP-interacting protein 1 (*CYFIP1*) is one of the four genes contained within this locus and has been associated with other genetic forms of autism spectrum disorders (ASD). In mice, *Cyfip1* haploinsufficiency leads to alteration of dendritic spine morphology and defects in synaptic plasticity, two pathophysiological hallmarks of mouse models of ASD. At the behavioral level, however, *Cyfip1* haploinsufficiency leads to minor phenotypes, not directly relevant for 15q11.2 deletion syndrome or ASD. A fundamental question is whether neuronal phenotypes caused by the mutation of *Cyfip1* are relevant for the human condition. Here, we describe a synaptic cluster of ASD-associated proteins centered on CYFIP1 and the adhesion protein Neuroligin-3. *Cyfip1* haploinsufficiency in mice led to decreased dendritic spine density and stability associated with social behavior and motor learning phenotypes. Behavioral training early in development resulted in alleviating the motor learning deficits caused by *Cyfip1* haploinsufficiency. Altogether, these data provide new insight into the neuronal and behavioral phenotypes caused by *Cyfip1* mutation and proof-of-concept for the development of a behavioral therapy to treat phenotypes associated with 15q11.2 syndromes and ASD.

## Introduction

Autism spectrum disorders (ASD) are defined by persistent deficits in social communication and social interaction accompanied by restricted, repetitive patterns of behavior, interests, or activities. In addition to these core symptoms, more than 70% of individuals with ASD have co-occurring medical, developmental, or psychiatric conditions^1^. An extensive variety of impairments affecting gross and fine motor functions affect more than 79% of individuals with ASD^1^. Despite the frequency of motor anomalies, studies on the underlying pathophysiology of motor deficits and their treatment remain limited.

Deletions in the 15q11.2 region of the human genome are associated with neurobehavioral deficits, and motor development delay and, in about 30% of individuals, symptoms of autism^2^. The 15q11.2 locus contains four genes, tubulin gamma complex-associated protein 3 (*TUBGCP3*), cytoplasmic FMR1 interacting protein 1 (CYFIP1), and non-imprinted in Prader-Willi/Angelman syndrome 1 and 2 (*NIPA1/2*)), and is also associated with larger 15q11–q13 type I deletions causing Prader-Willi or Angelman Syndromes^3^. Microdeletions of 15q11.2 correlated with decreased *TUBGCP3*, *CYFIP1*, and *NIPA1/2* mRNA levels in lymphoblastoid cells derived from patients with Prader-Willi syndrome^4^. The four genes in the 15q11.2 locus were shown to impact on the behavioral symptoms of patients with 15q11.2 deletion or Prader-Will syndrome. In addition, decreased *CYFIP1* mRNA levels were also found in leukocytes obtained from ASD diagnosed patients with Fragile X and in patients diagnosed with autism and carrying a deletion of SH3 and multiple ankyrin repeat domains 2 (SHANK2) gene.

Amongst the genes within region 15q11.2, *CYFIP1* is of particular interest due to its role in the control of actin dynamics^5,6^ and protein translation^7^. This regulation occurs through its interaction with the Wiskott–Aldrich syndrome protein family verprolin-homologous protein (WAVE) regulatory complex (WRC)^5,6^, and fragile X mental retardation protein (FMRP)^7^, respectively. Hippocampal neurons with Cyfip1 haploinsufficiency (*Cyfip1*^HET^) show defects in dendritic spine morphogenesis and increased metabotropic glutamate receptor-induced long-term depression^8,9^, two phenotypes consistently found in mouse models of ASD and in particular Fragile X syndrome^10–12^. Importantly, analyses of post-mortem tissue of individuals with idiopathic and syndromic forms of ASD also showed alteration of dendritic spine morphology^11^. These results show that the effects of deletion of *Cyfip1* resemble the cellular phenotypes found in other models of ASD. However, mice heterozygous for *Cyfip1* (*Cyfip1*^HET^) show mild behavioral phenotypes^8,13^. It is, therefore, possible that CYFIP1 function at synapses is not relevant for ASD or that the behavioral phenotyping of *Cyfip1*^HET^ mice was not conducted extensively enough.

Within the WRC, CYFIP1, and Abi interactor proteins 1–3 (ABI1–3) form an interaction surface that enables the interaction with proteins containing a WRC interacting receptor sequence (WIRS). The coding sequence of synaptic adhesion proteins Neuroligin-3 contains a WIRS and is therefore predicted to interact with CYFIP1 and ABI 1–3. The genes coding for these proteins have been associated with ASD in humans^14–19^. A putative interaction between Neuroligin-3, CYFIP1, and FMRP would point towards a possible common pathway linking ASD-associated proteins at synapses. Mice lacking Neuroligin-3 or FMRP show defects of social behavior and motor learning deficits correlated^20–25^ with alterations of dendritic spine stability^26,27^. Based on this predicted interaction, we hypothesized that *Cyfip1*^*Het*^ mice also show defects in social and motor behaviors. This study examines these possibilities by immunoprecipitation paired with mass-spectrometry, analysis of dendritic spine plasticity in vivo and behavioral analysis. Based on the results obtained, we investigated the possibility rescuing *Cyfip1*^HET^ mice motor learning phenotype using a behavioral approach.

## Results

**Fig. 1:**
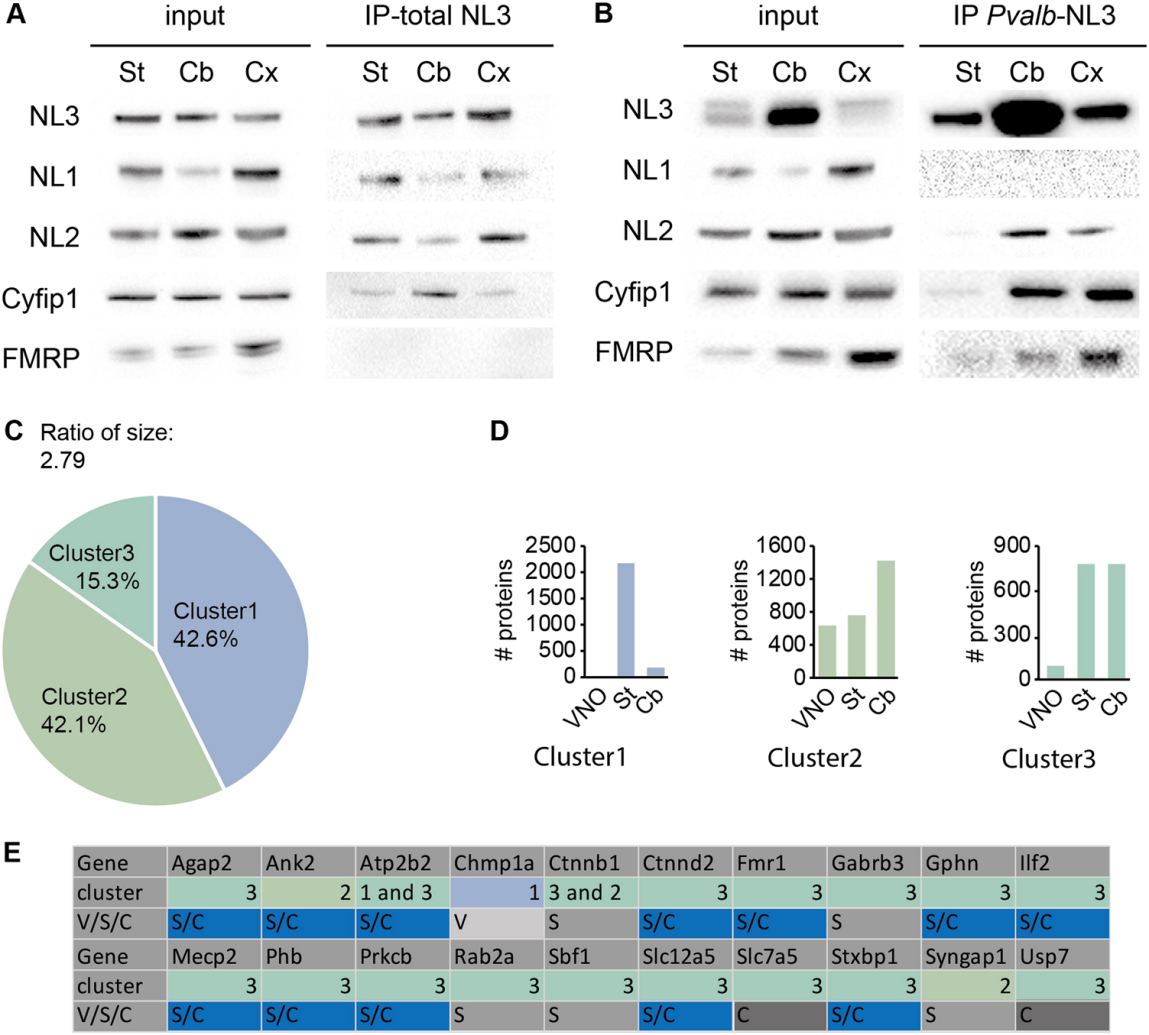
Binding between Neuroligin-3 and Cyfip1 in *Pvalb*-expressing neurons. **a** Western blot analysis showing that Neuroligin-1, 2, and 3 (NL1, 2, and 3) and Cyfip1 are detected in proteins co-immunoprecipitated with Neuroligin-3 in wild-type striatum (St), cerebellum (Cb), and cortex (Cx). Note that FMRP is not detected in co-immunoprecipitated proteins. **b** Western blot analysis showing that Neuroligin-2 (NL2), Cyfip1, and FMRP are detected in proteins co-immunoprecipitated with Neuroligin-3 in striatum (St), cerebellum (Cb), and cortex (Cx) of *Nlgn3*^KO^*Pvalb*^Cre^ mice. Note that Neuroligin-1 was not detected in co-immunoprecipitated proteins. **c** Two-step cluster analysis of proteins co-immunoprecipitated with Neuroligin-3 in wild-type mice VNO, *Nlgn3*^*KO*^*Pvalb*^Cre^ striatum (St), and cerebellum (Cb) but not in *Nlgn3*^KO^ mice VNO. **d** Bar graphs show the number of proteins per cluster and per anatomical region. **e** Details of the anatomical region (VNO (V), striatum (S), or cerebellum (C)) and cluster in which proteins coded by genes associated with ASD and co-immunoprecipitated with Neuroligin-3 are detected

The interaction between Neuroligin-3 and CYFIP1 was investigated using immunoprecipitation of Neuroligin-3 in striatum, cerebellum, and cortex, followed by western blot analysis. Neuroligin-3 co-immunoprecipitates Neuroligin-1 and 2, two previously characterized interactors of Neuroligin-3^28^, and CYFIP1 (Fig. 1a). CYFIP1 was detected in the three different brain regions indicating that the interaction between Neuroligin-3 and CYFIP1 is not restricted to a particular brain region. Note that FMRP was not detected in these samples. Further investigations revealed that CYFIP1 was also present in Neuroligin-3 immunoprecipitates from synaptosomes but again FMRP was not (Supplementary Figure 1a). Neuroligin-3 is expressed in neurons but also in astrocytes^29^. To determine if the interaction between Neuroligin-3 and CYFIP1 occurs in neurons, immunoprecipitations were performed on brain samples originating from mice expressing Neuroligin-3 only in *Pvalb*-expressing neurons (*Nlgn3*^KO^*Pvalb*^*Cre*^), as we previously showed that re-expressing Neuroligin-3 in these cells was sufficient to restore the ASD-related deficits in *Nlgn3* knockout mice^21^. Neuroligin-3 neuronal co-immunoprecipitated proteins from the three brain regions contained Neuroligin-2 and CYFIP1, indicating that indeed interaction between these proteins occurs in neurons and is not grossly influenced by the regional localization of *Pvalb*-expressing neurons (Fig. 1b and supplementary Fig. 1b). Interestingly, proteins from *Nlgn3*^KO^*Pvalb*^*Cre*^, co-immunoprecipitated with Neuroligin-3, did not contain Neuroligin-1 but did contain FMRP, suggesting that the identity of the proteins interacting with the Neuroligin-3/CYFIP1 complex vary depending on the cell type.

Studies suggest that CYFIP1's interaction with FMRP and WRC is mutually exclusive^7^. To determine if the Neuroligin-3/CYFIP1 complex interacts exclusively with FMRP or also contains components of the WRC we performed an unbiased screen of Neuroligin-3 co-immunoprecipitated proteins using mass-spectrometry. The synapse-specific interactome of Neuroligin-3 was investigated, in vivo, in *Nlgn3*^KO^*Pvalb*^Cre^ mice tissue (*N* = 3, supplementary Table 1), and in tissue expressing Neuroligin-3 but lacking synapses (*N* = 3, supplementary Table 2), as a control. The so-called olfactory ensheathing cells (OEC) populating the vomeronasal organ (VNO) fulfill these criteria as they are known to express Neuroligin-3^30^, but are entirely devoid of synapses. Note that, biochemical analysis and in situ hybridization demonstrated that Neuroligin-3 is expressed in non-neuronal cells in the VNO, confirming its expression in OEC (supplementary Fig. 2a–d). To determine proteins specifically associated with Neuroligin-3 in *Pvalb*-expressing interneurons, we performed co-immunoprecipitation of Neuroligin-3 coupled with mass-spectrometry analysis and included co-immunoprecipitation of the VNOs of wild-type (*N* = 3, supplementary Table 2) and *Nlgn3*^*KO*^ mice (*N* = 2) to our analysis to control for non-neuronal co-immunoprecipitated proteins and unspecific binding of the Neuroligin-3 antibody, respectively (Supplementary Tables 1 and 2). We used hierarchical cluster analysis of co-immunoprecipitated proteins in wild-type mice VNO or *Nlgn3*^*KO*^*Pvalb*^Cre^ mice striatum or cerebellum but not in *Nlgn3*^*KO*^ mice VNO to determine the optimal number of clusters in our dataset, which was found to be three. On this basis, follow-up two-step cluster analysis showed that clusters 1, 2, and 3 contained 42.6%, 42.1%, and 15.3% of co-immunoprecipitated proteins, respectively (Fig. 1c). Analysis of the number of co-immunoprecipitated proteins contained in each cluster revealed that cluster 1 proteins almost uniquely originated from the striata and cluster 2 proteins originated from the three anatomical regions (Fig. 1d). Interestingly, cluster 3 was mostly composed of proteins co-immunoprecipitated from striata and/or cerebella but not VNOs, suggesting the existence of proteins interacting with Neuroligin-3 specifically in neurons. Consistent with Neuroligin-3 synaptic localization in neurons, excitatory (PSD93 and 95 and synapse-associated protein 97 and 102), and inhibitory synaptic proteins (Neuroligin-2 and Neuroligin-4 and Gephyrin) were co-immunoprecipitated only in *Pvalb*-expressing neurons independently of the brain region (Supplementary Table 3). As FMRP, WAS protein family member 1 (WASF1) and ABI1 were detected in proteins immunoprecipitated by Neuroligin-3. FMRP was detected in *Nlgn3*^KO^*Pvalb*^Cre^ mice cerebella and striata, whereas WASF1 and ABI1 were only detected in striata (Supplementary Table 3). These results suggest that binding between Neuroligin-3 and CYFIP1 and WRC is influenced by the localization of the *Pvalb*-expressing cells (Supplementary Table 3). Interestingly, the protein products of genes reliably associated with syndromic and non-syndromic ASD (with an SFARI gene score of 1, 2, or 3) were also co-immunoprecipitated only in *Pvalb*-expressing interneurons, independently of the brain region (Fig. 1e). Altogether, these results show that Neuroligin-3 and CYFIP1 interact in neurons in vivo and that the identity of the proteins they interact with is influenced by the regional localization of the neurons. Moreover, they suggest that CYFIP1 may interact with other ASD-associated proteins at excitatory and inhibitory synapses, either directly or indirectly via its binding to Neuroligin-3 (Supplementary Fig. 1b).

**Fig. 2:**
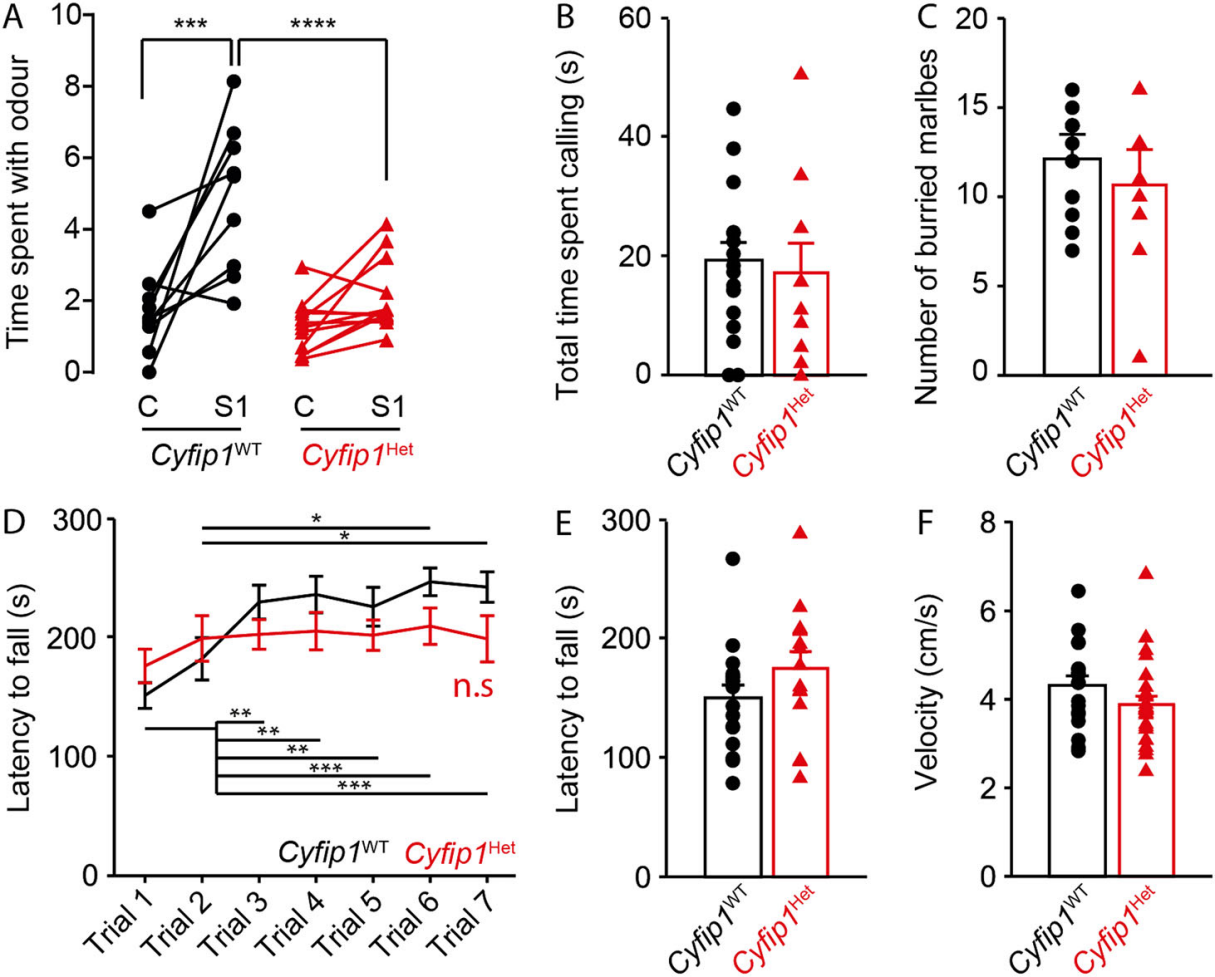
*Cyfip1* haploinsufficiency leads to motor learning defects. **a** *Cyfip1*^HET^ mice spent a similar amount of time sniffing control, non-social, odor (C) and social odor (S), whereas their *Cyfip1*^WT^ littermate spent more time sniffing the social odor (S) than the control one (C). **b** *Cyfip1*^HET^ mice and their *Cyfip1*^WT^ littermates spent a similar amount of time calling when exposed to females in estrus. **c** *Cyfip1*^HET^ mice and their *Cyfip1*^WT^ littermates buried a similar number of marbles. **d** The time *Cyfip1*^WT^ mice spent on an accelerating rotarod significantly increased over seven trials, whereas that of *Cyfip1*^HET^ did not. **e** *Cyfip1*^WT^ and *Cyfip1*^HET^ mice spent similar amounts of time on an accelerating rotarod on the first trial. **f** *Cyfip1*^WT^ and *Cyfip1*^HET^ mice’s velocity in the open field was similar. In (**a**) connected dots represent the same animal tested for the control (C) and the social (S) odors. All values in (**b**)–(**d**) are represented as mean ± SEM. In (**a**) and (**d**), statistical significance was tested by repeated measures ANOVA followed by Bonferroni Post-hoc test. *P ≤ 0.05; **P ≤ 0.01; ***P ≤ 0.001; and ****P ≤ 0.0001. In (**b**), (**c**), (**e**), and (**f**), statistical significance was tested by two-tailed Student’s *t*-test or one-tailed Mann–Whitney test

Mice lacking *Fmr1* or *Nlgn3* show behavioral phenotypes associated with the core symptoms of ASD, including defects in social behavior, but also with some of its comorbidities (e.g. hyperactivity, alteration of motor learning)^21,25,31–34^. We hypothesized that since CYFIP1 interacts with both Neuroligin-3 and FMRP, the behavioral phenotype should be similar across the three models. A complete absence of *Cyfip1* is embryonic lethal^7^, therefore we used a *Cyfip1*^HET^ mouse model. *Cyfip1*^HET^ mice showed an absence of interest for social cues compared to their *Cyfip1*^WT^ littermates (Fig. 2a) without deficits in social dominance (courtship vocalization test, Fig. 2b) or repetitive behaviors (marble burying test, Fig. 2c). In addition, *Cyfip1*^HET^ mice showed motor learning deficits, as indicated by the absence of an improved performance in the rotarod test, compared to their *Cyfip1*^WT^ littermates (Fig. 2d), but no change in basal motor activity (Fig. 2e, f). The biochemical characterization of *Cyfip1*^HET^ mice showed a significant decrease of CYFIP1 protein expression levels in motor and frontal cortex and in the hippocampus but no change in other brain regions or in the periphery (Supplementary Fig. 3). *Cyfip1*^HET^ mice phenotype is consistent with the region-specific decrease of CYFIP1 protein expression since frontal and motor areas of the cortex are involved in the control of social and motor functions. In addition, the behavioral phenotype of *Cyfip1*^HET^ mice is also consistent with the association between *CYFIP1* and 15q11.2 deletion syndrome and ASD in humans, therefore validating the use of the mouse model for ASD studies.

**Fig. 3:**
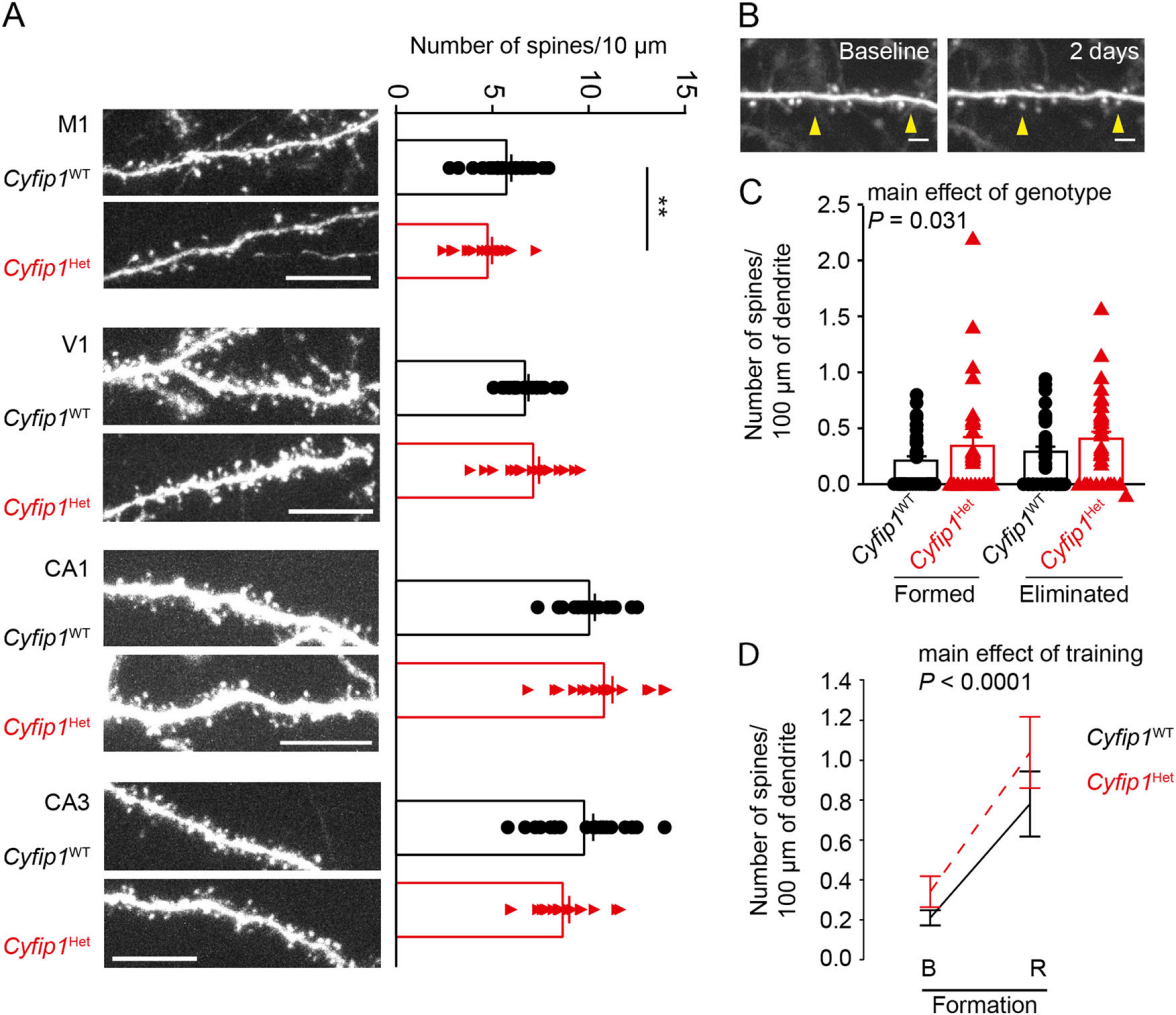
*Cyfip1* haploinsufficiency leads to decreased stability of dendritic spines without affecting their formation. **a** Dendrites of GFP-expressing neurons from the motor cortex (M1), visual cortex (V1), and hippocampus (CA1 and CA3) of *Cyfip1*^WT^ and *Cyfip1*^HET^ mice. Scale bars represent 10 μm. Quantification of spine density of *Cyfip1*^WT^ and *Cyfip1*^HET^ dendrites. **b** Two images of the same dendrite of a *Cyfip1*^WT^ mice motor neuron taken at an interval of 2 days showing the newly formed dendritic spines (arrows). Scale bars represent 2 μm. **c** Quantification of formed and eliminated dendritic spines in motor cortices of *Cyfip1*^WT^ and *Cyfip1*^HET^ mice. **d** Quantification of formed dendritic spines in motor cortices of *Cyfip1*^WT^ and *Cyfip1*^HET^ mice before (B) and after rotarod training (R). All values are represented as mean ± SEM. In (**b**) and (**d**), statistical significance was tested by ANOVA followed by Bonferroni post-hoc test. **P ≤ 0.01

*Cyfip1*^HET^ hippocampal neurons show deficits in dendritic spine maturation in vitro and ex vivo^9^, a converging cellular phenotype between syndromic and non-syndromic genetic mouse models of ASD^10,11^. Decreased dendritic spine maturation can be explained by defects in dendritic spine formation and/or dendritic spine instability. To investigate these two possibilities, we crossed *Cyfip1*^HET^ mice with mice expressing green fluorescent proteins in a subset of neurons (refer to Material and methods) and imaged dendritic spines of cortical neurons ex vivo and in vivo in adult male mice. Immunohistology experiments demonstrated that *Cyfip1*^HET^ mice showed decreased dendritic spine density in neurons from motor cortex but not from the V1 area of the visual cortex, compared to their *Cyfip1*^WT^ littermates (Fig. 3a). Two-photon imaging to track dendritic spines in the motor cortex in vivo revealed an increased formation and elimination of dendritic spines over time, in *Cyfip1*^HET^ mice, compared to *Cyfip1*^WT^ littermates (Fig. 3b, c). Since increased rotarod performance correlates with increased formation of dendritic spines^35^, we hypothesized that, in addition to increased dendritic spine instability, *Cyfip1*^HET^ mice show defects in dendritic spine formation. As previously demonstrated^35^, motor training increased the formation of dendritic spines by about three-fold in *Cyfip1*^WT^ mice without affecting the number of eliminated spines (Fig. 3d). Despite their lack of increased performance on the rotarod, *Cyfip1*^HET^ mice showed an increased number of newly formed dendritic spines upon motor training similar to that of their *Cyfip1*^WT^ littermates (Fig. 3d). Note that rotarod training had no effect on dendritic spine elimination in both genotypes. These results show that while *Cyfip1*^*HET*^ does not impact on the ability to form new spines it leads to an increased dendritic spines instability.

**Fig. 4:**
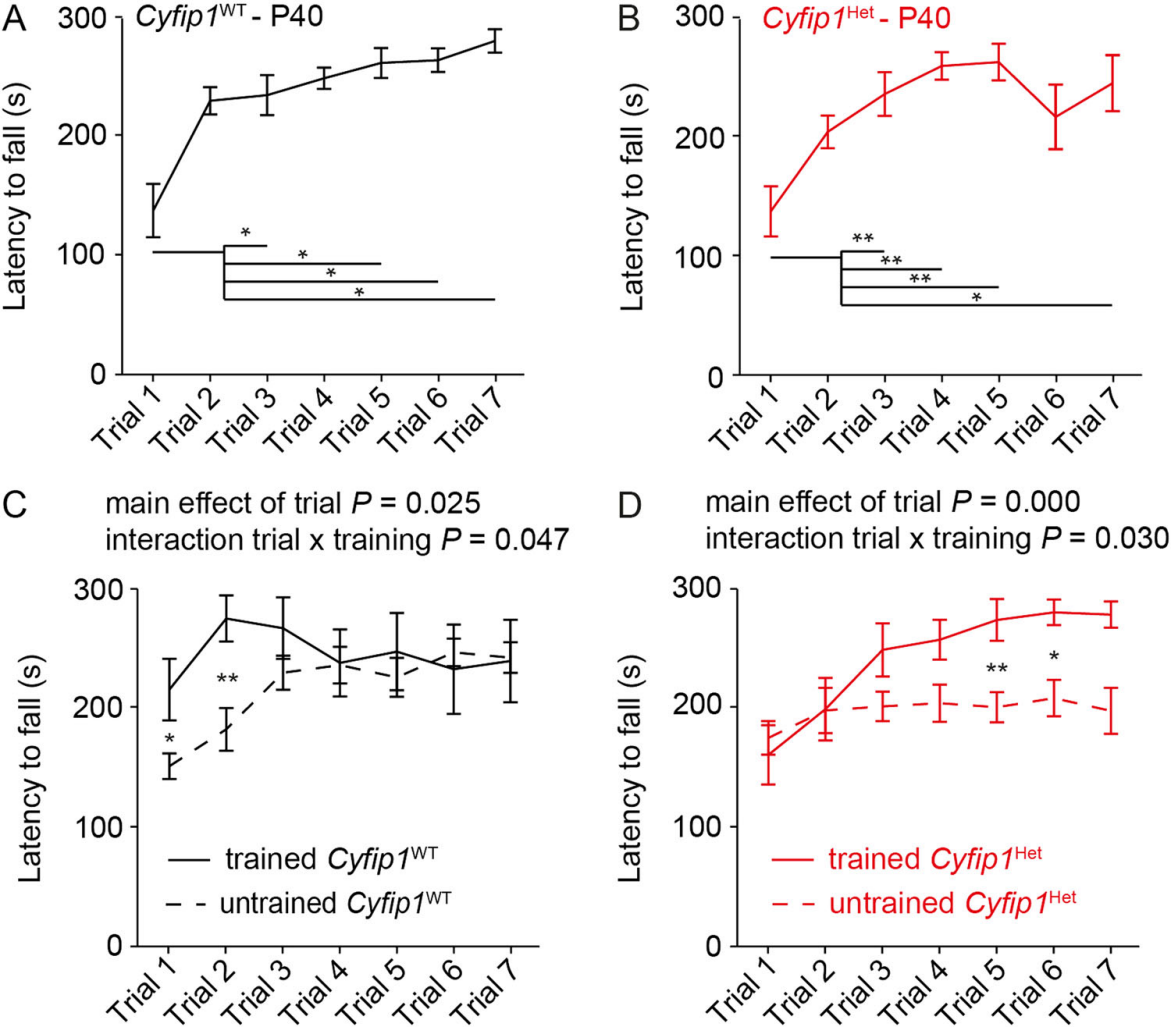
Motor training at early stages of development alleviates *Cyfip1*^HET^ motor deficits. **a**, **b** Wild-type and *Cyfip1*^HET^ male mice improved their latency to fall off the accelerating rod at P40. **c** Trained *Cyfip1*^WT^ mice showed increased motor performances during the first two trials during the behavioral assessment at P60 compared to untrained adult wild-type mice. **d** Trained *Cyfip1*^HET^ mice increased their motor performances during the rotarod testing compared to untrained *Cyfip1*^HET^ mice. All values presented as mean ± SEM. Statistical significance was tested by repeated measures ANOVA followed by Bonferroni post-hoc test. *P < 0.05; **P < 0.01; and ***P *<* 0.001

Given that *Cyfip1*^HET^ mice show a similar ability to form new spines upon training as *Cyfip1*^WT^ mice, we hypothesized that training *Cyfip1*^HET^ mice during development, before the appearance of the behavioral phenotype, would be sufficient to rescue their motor learning deficits. The earliest time point in the development animals complied with the motor learning protocol was postnatal day 40 (P40), when *Cyfip1*^WT^ mice show reliable decreased latency to fall from an accelerating rod over successive trials (Fig. 4a). At P40, *Cyfip1*^HET^ mice showed a decreased latency to fall from the accelerating rod over successive trials (Fig. 4b). These results indicate that *Cyifp1*^HET^ mice motor learning deficits develop after P40. To test our hypothesis we trained *Cyfip1*^WT^ and *Cyfip1*^HET^ mice at postnatal days 40, 50, and 51 using the same rotarod protocol (in the following referred to as ‘trained’ mice) and tested for their motor behavior at P60. Trained *Cyfip1*^WT^ mice showed an increased latency to fall from an accelerating rod at trials 1 and 2 and no difference at the subsequent trials compared to untrained *Cyfip1*^WT^ mice, indicating that, as previously demonstrated^35^, motor training increased the basal motor performance of wild-type mice (Fig. 4c). Trained *Cyfip1*^HET^ mice showed a similar latency to fall from an accelerating rod from trials 1 to 4 and subsequently increased latency to fall compared to untrained *Cyfip1*^HET^ mice, suggesting that sustained motor training during the development is sufficient to alleviate the motor deficits of adult *Cyfip1*^HET^ mice (Fig. 4d).

## Discussion

Three important conclusions can be drawn from this study. First, we found that CYFIP1 binds to Neuroligin-3 at synapses and, directly or indirectly, to other proteins associated with ASD. Second, we showed that *Cyfip1*^*HET*^ is associated with alterations of dendritic spine stability, but not formation, and defects in social behavior and motor learning. Finally, based on these findings we designed a behavioral approach that alleviated the motor learning phenotype of *Cyfip1*^HET^ mice.

### Dendritic spine defects as a converging phenotype in ASD

Previous work with genetic mouse models suggested that the different genetic mutations associated with ASD target a small set of molecular pathways, leading to a deficit in dendritic spine morphogenesis^10,11^. Our study is the first to provide direct evidence to support this hypothesis. Importantly, defects in dendritic spine morphology are also reported in human post-mortem studies, showing that this pathophysiological feature is one of the few conserved between mouse models and humans.

When studied in isolation, the impact of specific monogenic genetic risk factors for ASD is limited by the frequency of the mutations in the human population, which is, in general, very low^36^. Our results demonstrate that some of these risk factors are in fact directly connected, such that they are likely to form a single molecular pathway in which dysfunction can cause specific forms of ASD. The frequency of such molecular alterations is likely to be equivalent to the sum of the frequency of the genetic risks, thus accounting for a large number of individuals with ASD. It remains possible that independent pathways regulating dendritic spine formation and function may alter the composition of this cluster of ASD proteins, suggesting that, not all cases of ASD associated where such changes are present, can be attributed to a single cause. Nevertheless, this finding is likely to provide a framework to address the genetic heterogeneity associated with ASD.

As such, analysis of dendritic spine morphology or density cannot be conveniently used as a biomarker in the human population, as it requires access to brain tissue. In this context, our findings are more likely to represent a step towards a better definition of some of the biological basis of ASD, which in turn may lead to the identification of biological markers. Indeed, dendritic spines are the postsynaptic docking compartment for a large number of excitatory synapses and decrease in their number and stability is likely to have a direct consequence on the number of synaptic inputs a neuron receives and, by extension, a brain region receives. Such connectivity between brain regions can be quantified in mice and humans using diffusion tensor imaging. Using *Cyfip1*^Het^ mouse model, and by extension, any genetic model associated with the ASD protein cluster could, therefore, help to better understand the complex relationship between brain region-specific defects in dendritic spine stability and brain-wide connectivity providing an experimental framework to identify biological markers for specific behavioral phenotypes.

### Predicted functional consequences associated with the ASD cluster of proteins

Aberrant signaling through type 1 metabotropic glutamate receptors 1 and 5 (mGluR1 and 5), resulting in defects in long-term synaptic depression (LTD), is a pathophysiological hallmark of genetic mouse models of ASD^37^. Interestingly, mutations in genes associated with the proteins co-immunoprecipitated with Neuroligin-3, including Neuroligin-3 itself^32,38^ (but see ref. ^39^), CYFIP1^8^, FMRP^40^, MeCP2^41^, Synaptic Ras GTPase-activating protein 1 (encoded by the *Syngap1* gene)^42^, and the beta 3 subunit of Type A gamma-aminobutyric acid receptors (encoded by the *Gabrb3* gene)^43^, lead to such phenotypes. These results indicate that in addition to regulating dendritic spine morphogenesis, this cluster of proteins might be involved in regulation of mGluR1/5 function and control of LTD.

At synapses, mGluR1/5 binds directly to Homer proteins, which links the receptors to the endoplasmic reticulum (ER), via interactions with PI 3-kinase enhancer (PIKE) and inositol 1,4,5-trisphosphate receptors (IP3Rs), or to ionotropic glutamate receptors, via SH3 and multiple ankyrin repeat domains (Shank) proteins^44^. Our data show that type 1 mGluR, and mGluR1 specifically, are reliably co-immunoprecipitated with Neuroligin-3 in cerebellar but not striatal *Pvalb*-expressing cells (Supplementary Table 3). Moreover, along with mGluR1 we also reliably detected PIKE (encoded by the *Agap3* gene) and IP3R1 (encoded by the *Itpr1* gene) but not Shank proteins or ionotropic receptors (Supplementary Table 3). Interestingly, FMPR has been shown to inhibit PIKE and therefore type 1 mGluR downstream signaling^45^. These results raise the question of a potential involvement of CYFIP1 in regulating type 1 mGluR downstream signaling. It is possible that Neuroligin-3, FMRP, and CYFIP1 are expressed in different subcellular compartments of the neurons and, therefore, do not aggregate in a single cluster. Nevertheless, these results suggest that Neuroligin-3, FMRP, and CYFIP1 are involved in localizing mGluR1 and the ER at the synapse. The release of calcium from the ER to the cytoplasm is regulated by mGluR1 and essential for the induction of LTD^46^. Based on our results, we can further speculate that defects in mGluR-LTD observed in genetic mouse models for ASD result from an inappropriate localization of the mGlur/ER complex at the synapse, a hypothesis that remains to be experimentally tested. Importantly, this mechanism might be cell or type 1 mGluR specific as we did not reliably detect type 1 mGluR in proteins co-immunoprecipitated with Neuroligin-3 in the striatum.

### Behavioral treatment to alleviate ASD core and comorbid symptoms

About 42% of individuals carrying 15q11.2 deletions show motor delay^2^. Motor abnormalities and delay in motor development are common comorbid symptoms in more than 70% of individuals with ASD^1^. In this study, we found that adult *Cyfip1*^HET^ mice show defects in motor learning. Note that we have not fully characterized the motor learning deficits and it is possible that a less challenging paradigm, with longer interatrial intervals for example, would find milder or no phenotype in *Cyfip1*^HET^ mice. Nevertheless, along with *Cyfip1*^HET^ mice, other mouse models for ASD show motor phenotypes^47^, suggesting that, to a certain extent, mouse models of ASD replicate motor abnormalities found in humans. Importantly, we found that training *Cyfip1*^*HET*^ mice early in the development is sufficient to alleviate the motor learning phenotype in adult mice. These results demonstrate that the motor learning phenotype of *Cyfip1*^HET^ mice develops over time, appearing between young adult and adult stages, and is reversible using behavioral training approaches. As future studies, it would be interesting to know if dendritic spine phenotypes follow the similar developmental trajectories and sensitivity to behavioral training. These results provide important insights for the treatment of motor impairment in ASD in humans. In humans, motor impairments can be detected early in the development alongside the diagnosis of ASD. Our results suggest that early behavioral intervention, targeted at stimulating motor function, might help in preventing motor impairment arising later in life.

## Materials and methods

### Animals

All procedures were conducted in accordance with the Animals (Scientific Procedures) Act 1986 (amended 2012). *Nlgn3*^*KO*^ mice^48^ were crossed with *Omp*^*Cre*^ mice (JAX: #006668^49^), where part of the *Omp* gene was replaced by Cre recombinase. *Nlgn3*^*KO*^ mice were also crossed with *Pvalb*^*Cre*^ mice, expressing Cre recombinase under the *Pvalb* promoter (JAX:017320^50^). The *Cyfip1*^tm2a(EUCOMM)Wtsi^ (EUCOMM) mouse line was either bred with BL6 mice to obtain *Cyfip1*^Het^ and *Cyfip1*^WT^ mice or crossed with Tg(Thy1-EGFP)MJrs/J (JAX: # 007788^51^) to obtain Thy1-EGFP-*Cyfip1*^Het^ and Thy1-EGFP-*Cyfip1*^WT^ mice. Mice were kept on a 12 h light/dark cycle with free access to food and water. All behaviors were assessed during the light cycle and after mice were habituated for 30 min to the testing room.

### Biochemistry

Brain tissue from adult mice was sliced in 1.5–2 mm sections and micro-dissected prior to snap freezing and storage at −80 °C. 10 µl of lysis buffer (1% SDS, 10 mM hepes, protease inhibitors, phosphatase inhibitors, 1 mM NaF, 1 mM NaVO_4_) was used per 1 mg of tissue. Using Laemmli buffer samples were run on SDS–PAGE gels (NuPAGE Novex 4–12% Bis–Tris, Protein gels, Invitrogen) and transferred on nitrocellulose membrane. Immunoblotting was performed with antibodies raised against CYFIP1 (anti-CYFIP1 ab156016, Abcam), β-III Tubulin (801201, BioLegend), and GAPDH (83956, Abcam). Blots were developed using ECL (Santa Cruz and Advansta). Blots were digitally acquired by ChemiDoc (Biorad) and quantified using Image Lab Software (Biorad).

For synaptosome fractionation, mice were culled by cervical dislocation, and the brains were immediately dissected and homogenized in SynPer (Sigma-Aldrich) containing protease cocktail inhibitors (Sigma-Aldrich) and phosphatase inhibitors (10 mM NaF, 1 mM NaVO_4_). Homogenized tissues were then centrifuged to give pellets, resuspended in SynPer to obtain nuclear fractions, and supernatants (i.e. homogenates). Part of the supernatants was further centrifuged to give pellets, further resuspended in SynPer^®^ to give synaptosome fractions and cytosolic fractions in supernatants. The samples were then immediately processed for western blot analysis, or immediately used for immunoprecipitation experiments.

For immunoprecipitation, tissues were dissected and then homogenized in 100 ml of lysis buffer for each 10 mg of tissue (20 mM Tris–HCl, 150 mM NaCl, 1 mM EDTA, 1% Triton-X100, 10 mM NaF, 1 mM Na3VO_4_, protease inhibitor cocktail (Sigma-Aldrich)). The samples were centrifuged at 14,000 × *g* for 10 min at 4 °C and the supernatant containing the proteins was removed. The ethanol was removed from Sepharose beads coated with Protein A (GE Healthcare Life Sciences) by two washes with ice-cold phosphate-buffered saline, at 2000 × *g* for 2 min. The supernatant was pre-cleared by incubating with Protein A-Sepharose beads for 30 min at 4 °C. Protein A-Sepharose beads for immunoprecipitation were washed with lysis buffer at 2000 × *g* for 2 min. Proteins were then incubated for 2 h at 4 °C with Protein A-Sepharose beads and Neuroligin-3 antibody (#129311, Synaptic Systems, 1:1000). Unbound proteins were removed by three washes with lysis buffer. Peptides were then eluted in lithium dodecyl sulfate buffer (106 mM Tris–HCl, 141 mM Tris-base, 2% LDS, 10% glycerol, 0.51 mM EDTA, 0.22 mM Brilliant Blue, 0.175 mM Phenol Red, 10 mM DTT) at 70 °C for 10 min. Samples were frozen at −20 °C for storage.

### Mass-spectrometry analysis

Mass-spectrometry analysis was performed by the University of Bristol Proteomics Facility. Each sample was separated as a lane on an SDS–PAGE gel and electrophoresis was performed until the samples had run ~3 cm into the separating gel. The gel lane was then cut into three slices and each slice subjected to in-gel tryptic digestion using a DigestPro automated digestion unit (Intavis Ltd.). The resulting peptides were fractionated using an Ultimate 3000 nano-LC system in line with an LTQ-Orbitrap Velos mass spectrometer (Thermo Scientific). In brief, peptides in 1% (vol/vol) formic acid was injected onto an Acclaim PepMap C18 nano-trap column (Thermo Scientific). After washing with 0.5% (vol/vol) acetonitrile 0.1% (vol/vol) formic acid peptides were resolved on a 250 mm × 75 μm Acclaim PepMap C18 reverse phase analytical column (Thermo Scientific) over a 150 min organic gradient, using seven gradient segments (1–6% solvent B over 1 min, 6–15% solvent B over 58 min, 15–32% solvent B over 58 min, 32–40% solvent B over 5 min, 40–90% solvent B over 1 min, held at 90% solvent B for 6 min and then reduced to 1% solvent B over 1 min) with a flow rate of 300 ml min^−1^. Solvent A was 0.1% formic acid and Solvent B was aqueous 80% acetonitrile in 0.1% formic acid. Peptides were ionized by nano-electrospray ionization at 2.1 kV using a stainless-steel emitter with an internal diameter of 30 μm (Thermo Scientific) and a capillary temperature of 250 °C. Tandem mass spectra were acquired using an LTQ- Orbitrap Velos mass spectrometer controlled by Xcalibur 2.1 software (Thermo Scientific) and operated in data-dependent acquisition mode. The Orbitrap was set to analyze the survey scans at 60,000 resolution (at *m*/*z* 400) in the mass range *m*/*z* 300–2000 and the top 20 multiply charged ions in each duty cycle selected for MS/MS in the LTQ linear ion trap. Charge state filtering, where unassigned precursor ions were not selected for fragmentation, and dynamic exclusion (repeat count, 1; repeat duration, 30 s; exclusion list size, 500) were used. Fragmentation conditions in the LTQ were as follows: normalized collision energy, 40%; activation *q*, 0.25; activation time 10 ms; and minimum ion selection intensity, 500 counts.

The raw data files were processed and quantified using Proteome Discoverer software v1.4 (Thermo Scientific) and searched against the UniProt Mouse database (78740 sequences) using the SEQUEST algorithm. Peptide precursor mass tolerance was set at 10 ppm, and MS/MS tolerance was set at 0.8 Da. Search criteria included carbamidomethylation of cysteine (+57.0214) as a fixed modification and oxidation of methionine (+15.9949) as a variable modification. Searches were performed with full tryptic digestion and a maximum of one missed cleavage was allowed. The reverse database search option was enabled and all peptide data was filtered to satisfy false discovery rate (FDR) of 5%.

### Social interest

Social odors originated from two cages of three C57Bl/6 male mice with the different parental origins, maintained for 6 days with the same home cage bedding to allow for a concentration of odorants. Before the test, swabs were wiped in a zig-zag pattern across the bottom surface of the cage to collect the olfactory cues. Mice were acclimatized for 30 min to the presence of a cotton swab prior to testing. Mice were then exposed to a cotton swab without odor followed by a cotton swab with a social odor. During the 2 min exploration periods, the time spent sniffing the swab on the first exposure to each odor was recorded manually and blind to the genotype.

### Vocalization

We recorded ultrasonic vocalization (USV) emitted by the male mice exposed to a female mouse in estrus (1, 2). All male mice were first habituated for 3 min to the arena. Following the habituation, an unfamiliar female mouse in estrus was added to the same arena for 3 min. During this time the mice could interact with each other freely in the arena. The male vocalization behavior was recorded by a preamplifier (UltraSoundGate 416 H, Avisoft Bioacoustics) connected microphone (UltraSoundGate CM16, Avisoft Bioacoustics) placed above the arena. As previously described frequencies were recorded and analyzed for the total number of emitted calls and the total time spent calling using SASLabPro (Avisoft Bioacoustics).

### Marble burying

Mice were in individual cages (28 cm × 17 cm) each containing 20 marbles arranged in five rows of four marbles on top of 4 cm deep bedding over the time course of 30 min. The testing room used was dimly lit, with equal light distribution for all mice in the trial. A marble was buried when ≤50% of the marble was not visible. For counting and quantification, experimenters were blind to the genotype.

### Rotarod behavior

Prior to the experiment mice were handled over the course of 2 days. During a trial, the rod accelerated to 40 rpm within 5 min. The latency to fall was evaluated across seven subsequent trials with a rest period of 5 min in between. The latency to fall was determined based on the time spent on the rod until the test mouse fell off, gripped to the rod and followed the rod for a full rotation or the testing trial ended after 5 min.

### Activity and anxiety behavior

Spontaneous activity of the mice was tested in an open field arena (40 cm × 40 cm) for 20 min, in the dark. The mice were able to freely move and explore the environment during the test. The behavior was recorded by a computer linked video camera located above the arena. The arena was illuminated from the bottom by an infrared light source, for detection of the mice. EthoVision XT (Noldus) software was used for tracking and quantification of the average velocity.

### Immunohistology

Thy-1 GFP-expressing *Cyfip1*^WT^ and *Cyfip1*^HET^ mice were anesthetized and perfused with 4% paraformaldehyde in 0.1 M phosphate buffer (PB). Brains were post-fixed overnight, stored in OCT at −80 °C, then cut coronally into 50 μm sections on a cryostat (Leica Biosystems, Germany) and mounted on a series of glass slides. Confocal images were acquired on a Zeiss LSM700 upright confocal microscope (Carl Zeiss, Welwyn Garden City, UK), using a ×43 water immersion lens (NA = 1.3). At least 10 Z-stack images (2048 × 2048 pixels) spaced 0.5 μm apart were acquired in the primary motor and visual cortices of each animal, focusing on secondary branching dendrites from cells. Series stacks were reconstructed into two dimensions according to Z-stack projections of maximum intensity in ImageJ (NIH, USA, public domain). All images were coded at the time of acquisition and analyzed blinded to the experimental condition. Spines were manually identified and counted on 60–150 μm long dendrites, with *N* = 24 dendrites (from four mice) per condition analyzed. Spine density was calculated as the number of spines per 10 μm of dendrites.

### Structural plasticity imaging in vivo

Dendritic spine imaging was performed in awake and head fixed Thy-1 GFP-expressing *Cyfip1*^WT^ and *Cyfip1*^HET^ adult male mice (from postnatal day 60) with implanted cranial windows. Aseptic surgical procedures were conducted based on previously described protocols^35^. Approximately one hour prior to cranial window surgery, animals were administered with the antibiotic Baytril (5 mg/kg, s.c.) and the anti-inflammatory drugs carprofen (5 mg/kg, s.c.) and dexamethasone (0.15 mg/kg, i.m.). Anesthesia was induced then maintained using isoflurane at concentrations of 4%, then 1.5–2%, respectively. After animals were secured in a stereotaxic frame, the scalp and periosteum were removed from the dorsal surface of the skull, and a custom head plate was attached to the cranium using dental cement (Super Bond C&B), with an aperture approximately centered over right M1. A 3 mm circular craniotomy was next performed, centered over the forelimb area using stereotaxic coordinates 1.3 mm anterior to the bregma and 1.2 mm lateral from the midline. The craniotomy was then closed with a glass insert constructed from three layers of circular no. 1 thickness glass (1 × 5 mm, 2 × 3 mm diameter) bonded together with an optical adhesive (Norland Products; catalog no. 7106). Mice were imaged one-week post-surgery. Initial dendritic spine imaging was followed up after 2, 7, and 9 days*.* In vivo 2-photon imaging was performed using a resonant scanning microscope (Thorlabs, B-Scope) with a ×16 0.8NA objective with 3 mm working distance (Nikon). eGFP was excited at 980 nm using a Ti:sapphire laser (Coherent, Chameleon) with a maximum laser power at the sample of 20 mW. Z stacks were acquired at a frame rate of ~30 Hz, with 20 frames per depth, from 15 depths spaced by 2 µm. Imaged stretches of dendrite close to parallel to the imaging plane were selected based on an eGFP expression which was traced back to the soma located in layer 5 (*Z*: 400–500 µm from brain surface). Cortical surface vascular landmarks were used to locate the same stretches of dendrite between sessions. During two-photon imaging animals were free to run on a custom designed fixed axis cylindrical treadmill, and data collection was limited to stationary periods to avoid locomotion related brain movement. Imaging data were acquired using Scanimage 4.1 (HHMI Janelia). Imaging data were first corrected for brain motion using an automated rigid registration algorithm implemented in Matlab (MathWorks). The 20 frames from each depth were then averaged and a maximum intensity projection calculated over the *z* planes which encompassed the stretch of the dendrite of interest. Formed and eliminated spines were manually counted and normalized to 100 µm of dendrite (Thy1-EGFP-*Cyfip1*^Het^ *N* = 36, from four mice; Thy1-EGFP-*Cyfip1*^WT^ *N* = 40, from four mice).

### Statistical analysis

Prior the cluster analysis, every protein detected in immunoprecipitates from VNO of *Nlgn3*^KO^ mice (Supplementary Table 2) was filtered out of the ones immunoprecipitated from wild-type mice VNO (Supplementary Table 2) and *Nlgn3*^KO^*Pvalb*^Cre^ striatum and cerebellum (Supplementary Table 1), because proteins present in *Nlgn3*^KO^ are likely to be false positives. The list of remaining proteins can be found in Supplementary Table 3. A protein was considered detected when its score was >0, therefore obtaining a binarized dataset containing proteins detected (score of 1) and non-detected (score of 0) for each technical triplicate and each anatomical region. We used SPSS Statistics^®^ software to perform hierarchical cluster analysis based on the binarized score and determined that three clusters described the dataset best. We then performed a two-step analysis with three clusters and obtained the results presented in Fig. 1.

Supplementary Table 4 contains the data structure, type of test used, observed power and N for Figs. 3 and 4. Each test included enough animals to reach a power close to or above 0.8. We designed our tests groups to have mice from the different groups tested at the same time. We used SPSS Statistics^®^ software to systematically test for normality using Levene’s test. No animals were removed from the analyses. Pairwise comparisons were analyzed by two-tailed Student’s *t*-test for normally distributed datasets or two-tailed Mann–Whitney’s test for non-normally distributed datasets. All datasets used for two-way non-repeated and repeated measure ANOVAs were normally distributed and, when appropriate, followed by post-hoc Bonferroni’s test.

## Supplementary information

Supplementary Figure 1

Supplementary Figure 2

Supplementary Figure 3

Supplementary Table 1

Supplementary Table 2

Supplementary Table 3

Supplementary Table 4

Supplementary figures legend

## References

[1] Lai MC, Lombardo MV, Baron-Cohen S (2014). Autism. Lancet.

[2] Cox DM, Butler MG (2015). The 15q11.2 BP1-BP2 microdeletion syndrome: a review. Int. J. Mol. Sci..

[3] Butler MG, Bittel DC, Kibiryeva N, Talebizadeh Z, Thompson T (2004). Behavioral differences among subjects with Prader-Willi syndrome and type I or type II deletion and maternal disomy. Pediatrics.

[4] Bittel DC, Kibiryeva N, Butler MG (2006). Expression of 4 genes between chromosome 15 breakpoints 1 and 2 and behavioral outcomes in Prader-Willi syndrome. Pediatrics.

[5] Chen B (2014). The WAVE regulatory complex links diverse receptors to the actin cytoskeleton. Cell.

[6] Chen Z (2010). Structure and control of the actin regulatory WAVE complex. Nature.

[7] De Rubeis S (2013). CYFIP1 coordinates mRNA translation and cytoskeleton remodeling to ensure proper dendritic spine formation. Neuron.

[8] Bozdagi O (2012). Haploinsufficiency of Cyfip1 produces fragile X-like phenotypes in mice. PLoS One.

[9] Pathania M (2014). The autism and schizophrenia associated gene CYFIP1 is critical for the maintenance of dendritic complexity and the stabilization of mature spines. Transl. Psychiatry.

[10] Forrest MP, Parnell E, Penzes P (2018). Dendritic structural plasticity and neuropsychiatric disease. Nat. Rev. Neurosci..

[11] Phillips M, Pozzo-Miller L (2015). Dendritic spine dysgenesis in autism related disorders. Neurosci. Lett..

[12] Auerbach BD, Osterweil EKampBearMF (2011). Mutations causing syndromic autism define an axis of synaptic pathophysiology. Nature.

[13] Chung L (2015). Parental origin impairment of synaptic functions and behaviors in cytoplasmic FMRP interacting protein 1 (Cyfip1) deficient mice. Brain Res..

[14] Jamain S (2003). Mutations of the X-linked genes encoding neuroligins NLGN3 and NLGN4 are associated with autism. Nat. Genet..

[15] Glessner JT (2009). Autism genome-wide copy number variation reveals ubiquitin and neuronal genes. Nature.

[16] Ylisaukko-oja T (2005). Analysis of four neuroligin genes as candidates for autism. Eur. J. Hum. Genet..

[17] Sanders SJ (2011). Multiple recurrent de novo CNVs, including duplications of the 7q11.23 Williams syndrome region, are strongly associated with autism. Neuron.

[18] Levy D (2011). Rare de novo and transmitted copy-number variation in autistic spectrum disorders. Neuron.

[19] CY RK (2017). Whole genome sequencing resource identifies 18 new candidate genes for autism spectrum disorder. Nat. Neurosci..

[20] Radyushkin K (2009). Neuroligin-3-deficient mice: model of a monogenic heritable form of autism with an olfactory deficit. Genes Brain Behav..

[21] Kalbassi, S., Bachmann, S. O., Cross, E., Roberton, V. H., Baudouin, S. J. Male and female mice lacking neuroligin-3 modify the behavior of their wild-type littermates. e*Neuro* **4**, eneuro. 0145–17 (2017). 10.1523/ENEURO.0145-17.2017 PMC5548363 28795135

[22] Tabuchi K (2007). Neuroligin-3 mutation implicated in autism increases inhibitory synaptic transmission in mice. Science.

[23] Jaramillo TC, Liu S, Pettersen A, Birnbaum SG, Powell CM (2014). Autism-related neuroligin-3 mutation alters social behavior and spatial learning. Autism Res..

[24] Chadman KK (2008). Minimal aberrant behavioral phenotypes of neuroligin-3 R451C knockin mice. Autism Res..

[25] Michalon A (2012). Chronic pharmacological mGlu5 inhibition corrects fragile X in adult mice. Neuron.

[26] Isshiki M (2014). Enhanced synapse remodelling as a common phenotype in mouse models of autism. Nat. Commun..

[27] Wijetunge LS, Angibaud J, Frick A, Kind PC, Nagerl UV (2014). Stimulated emission depletion (STED) microscopy reveals nanoscale defects in the developmental trajectory of dendritic spine morphogenesis in a mouse model of fragile X syndrome. J. Neurosci..

[28] Sudhof TC (2008). Neuroligins and neurexins link synaptic function to cognitive disease. Nature.

[29] Stogsdill JA (2017). Astrocytic neuroligins control astrocyte morphogenesis and synaptogenesis. Nature.

[30] Gilbert M, Smith J, Roskams AJ, Auld VJ (2001). Neuroligin 3 is a vertebrate gliotactin expressed in the olfactory ensheathing glia, a growth-promoting class of macroglia. Glia.

[31] Rothwell PE (2014). Autism-associated neuroligin-3 mutations commonly impair striatal circuits to boost repetitive behaviors. Cell.

[32] Baudouin SJ (2012). Shared synaptic pathophysiology in syndromic and nonsyndromic rodent models of autism. Science (N. Y., NY).

[33] Koekkoek SKE (2005). Deletion of FMR1 in Purkinje cells enhances parallel fiber LTD, enlarges spines, and attenuates cerebellar eyelid conditioning in fragile X syndrome. Neuron.

[34] Roy S (2011). Comprehensive motor testing in Fmr1-KO mice exposes temporal defects in oromotor coordination. Behav. Neurosci..

[35] Yang G, Pan F, Gan WB (2009). Stably maintained dendritic spines are associated with lifelong memories. Nature.

[36] Spooren W, Lindemann L, Ghosh A, Santarelli L (2012). Synapse dysfunction in autism: a molecular medicine approach to drug discovery in neurodevelopmental disorders. Trends Pharmacol. Sci..

[37] D’Antoni S (2014). Dysregulation of group-I metabotropic glutamate (mGlu) receptor mediated signalling in disorders associated with Intellectual Disability and Autism. Neurosci. Biobehav. Rev..

[38] Martella, G. et al. The neurobiological bases of autism spectrum disorders: the R451C-neuroligin 3 mutation hampers the expression of long-term synaptic depression in the dorsal striatum. *Eur. J. Neurosci.* **47**, 701–708 (2017). 10.1111/ejn.13705 28921757

[39] Zhang B (2015). Neuroligins sculpt cerebellar Purkinje-cell circuits by differential control of distinct classes of synapses. Neuron.

[40] Bear, M. F., Huber, K. M., Warren, S. T. The mGIuR theory of fragile X mental retardation. *Trends Neurosci.* **27**, 370–377 (2004). 10.1016/j.tins.2004.04.009 15219735

[41] Gogliotti RG (2016). mGlu5 positive allosteric modulation normalizes synaptic plasticity defects and motor phenotypes in a mouse model of Rett syndrome. Hum. Mol. Genet..

[42] Barnes SA (2015). Convergence of hippocampal pathophysiology in syngap + /- and Fmr1-/y mice. J. Neurosci..

[43] Mercer, A. A., Palarz, K. J., Tabatadze, N., Woolley, C. S., Raman, I. M. Sex differences in cerebellar synaptic transmission and sex-specific responses to autism-linked Gabrb3 mutations in mice. *eLife* **5**, e07596 (2016). 10.7554/eLife.07596 PMC4878876 27077953

[44] Shiraishi-Yamaguchi Y, Furuichi T (2007). The Homer family proteins. Genome Biol..

[45] Sharma A (2010). Dysregulation of mTOR signaling in fragile X syndrome. J. Neurosci..

[46] Kano M, Hashimoto K, Tabata T (2008). Type-1 metabotropic glutamate receptor in cerebellar Purkinje cells: a key molecule responsible for long-term depression, endocannabinoid signalling and synapse elimination. Philos. Trans. R. Soc. B.

[47] Schroeder JC, Reim D, Boeckers TM, Schmeisser MJ (2017). Genetic animal models for autism spectrum disorder. Curr. Top. Behav. Neurosci..

[48] Tanaka KF (2010). Flexible accelerated STOP tetracycline operator-knockin (FAST): a versatile and efficient new gene modulating system. Biol. Psychiatry.

[49] Li J, Ishii T, Feinstein P, Mombaerts P (2004). Odorant receptor gene choice is reset by nuclear transfer from mouse olfactory sensory neurons. Nature.

[50] Hippenmeyer S (2005). A developmental switch in the response of DRG neurons to ETS transcription factor signaling. PLoS Biol..

[51] Feng G (2000). Imaging neuronal subsets in transgenic mice expressing multiple spectral variants of GFP. Neuron.

