# Supplementary figures and images for: Behavioral training rescues motor deficits in Cyfip1 haploinsufficiency mouse model of autism spectrum disorders
Source: Transl Psychiatry. 2019 Jan 21;9:29. doi: 10.1038/s41398-018-0338-9 (PMC6341103; doi:10.1038/s41398-018-0338-9)

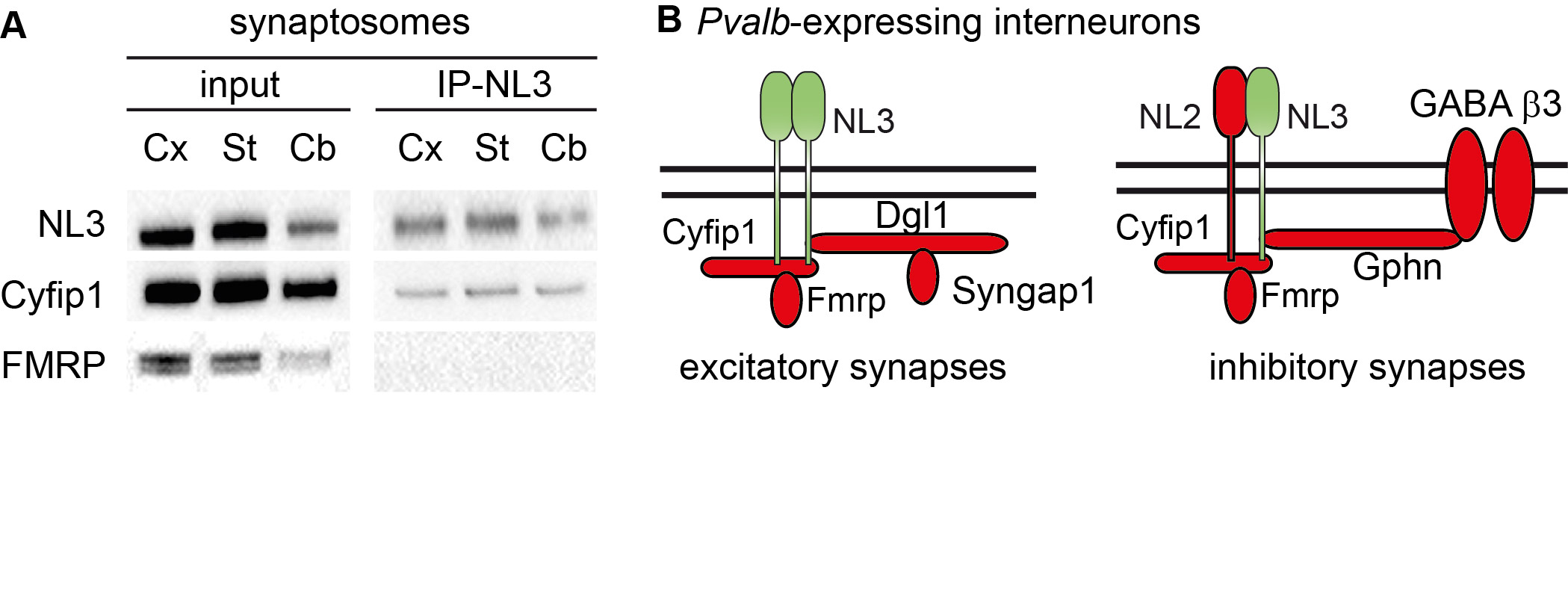

Supplement: Supplementary file 1 — Supplementary Figure 1 [file 41398_2018_338_MOESM1_ESM.jpg]

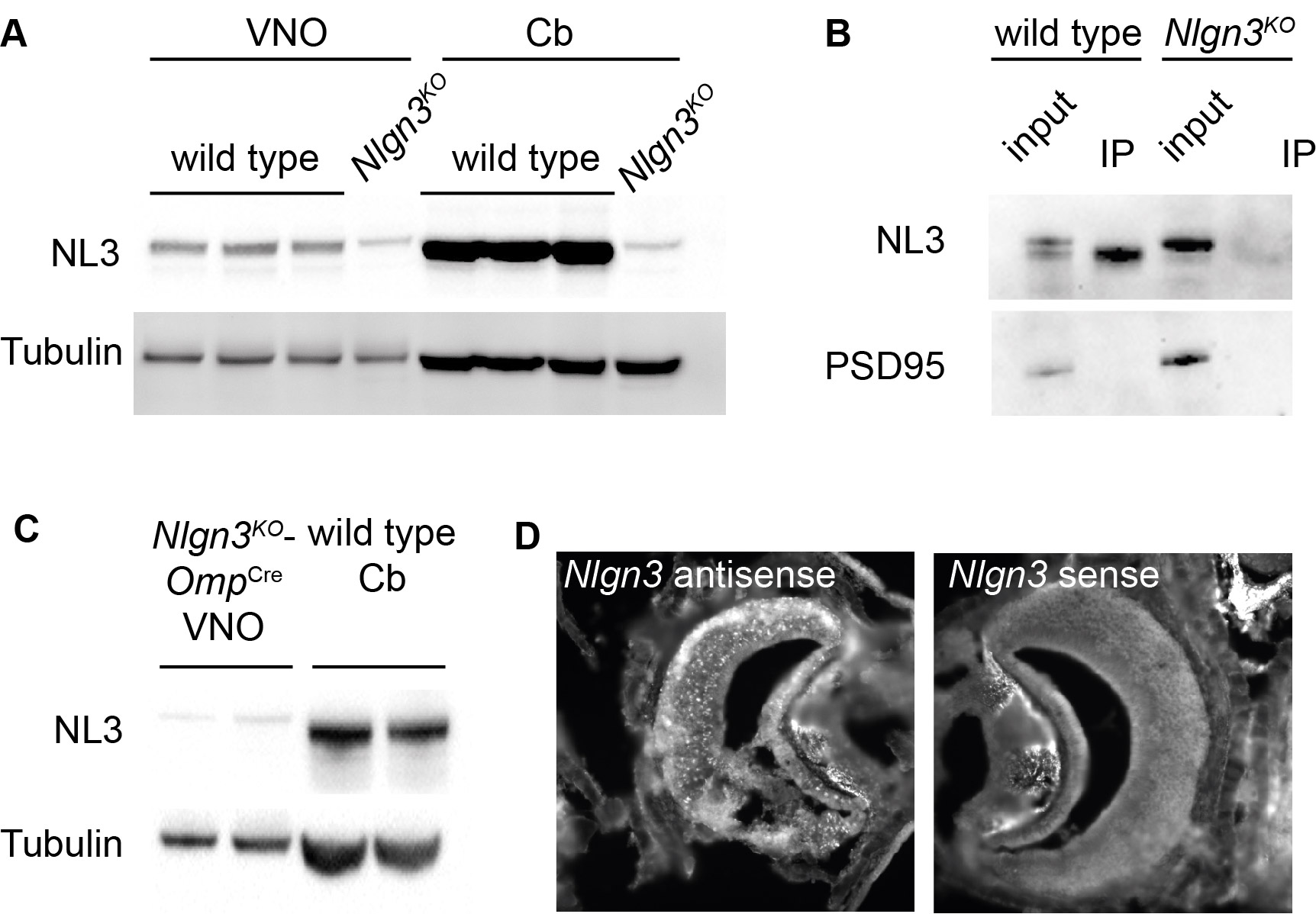

Supplement: Supplementary file 2 — Supplementary Figure 2 [file 41398_2018_338_MOESM2_ESM.jpg]

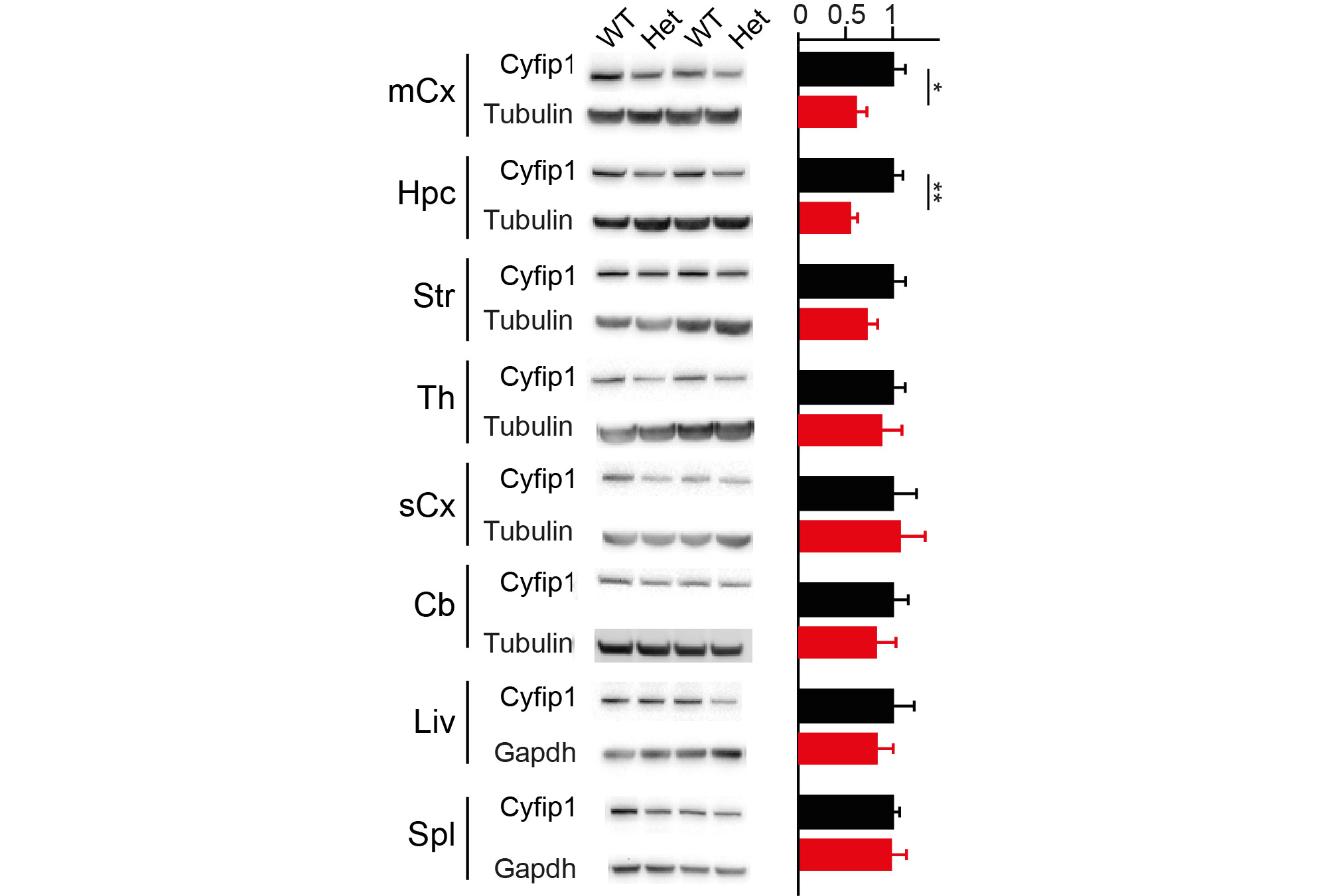

Supplement: Supplementary file 3 — Supplementary Figure 3 [file 41398_2018_338_MOESM3_ESM.jpg]
