# Supplementary material for: Behavioral training rescues motor deficits in Cyfip1 haploinsufficiency mouse model of autism spectrum disorders
Source: Transl Psychiatry. 2019 Jan 21;9:29. doi: 10.1038/s41398-018-0338-9 (PMC6341103; doi:10.1038/s41398-018-0338-9)
Supplement: Supplementary file 7 — Supplementary Table 4 [file 41398_2018_338_MOESM7_ESM.docx]

| **Figure** | **Shapiro-Wilk test**  **of normality** | **Test for**  **Equality of variances** | **Type of test** | **Observed power** | **Sample size** |
| --- | --- | --- | --- | --- | --- |
| **Figure 2 A** | *Cyfip1*^WT^  Control: *W* = 0.877, *P* = 0.176  S1: *W* = 0.862, *P* = 0.125  *Cyfip1*^HET^  Control: *W* = 0.901, *P* = 0.225  S1: *W* = 0.897, *P* = 0.203 | Mauchly`s Test of Sphericity  *W* = 1,  *χ^2^* = 0,  *P* = 0  Greenhouse-Geisser ε = 1 | Repeated measures ANOVA  Greenhouse-Geisser  Main effect of odour  *F_(1,19)_* = 23.43, *P* < 0.0001  Interaction odour x genotype  *F_(1,19)_* = 7.963, *P* = 0.011  Followed by Bonferroni corrected pairwise comparison | 0.996  0.763 | *Cyfip1*^WT^ n = 9,  *Cyfip1*^HET^ n = 12 |
| **Figure 2B**, Vocalisation | *Cyfip1*^WT^ *W* = 0.969, *P* = 0.770  *Cyfip1*^HET^*W* = 0.898, *P* = 0.207 | Levene`s test  *F* = 0.367, *P* = 0.550 | Two-tailed Student`s *t* test  *P* = 0.698 | NA | *Cyfip1*^WT^ n = 18,  *Cyfip1*^HET^n = 10 |
| **Figure 2C**, Marble burying | *Cyfip1*^WT^ *W* = 0.917, *P* = 0.173  *Cyfip1*^HET^*W* = 0.896, *P* = 0.083 | Levene`s test  *F* = 0.502, *P* = 0.485 | Two-tailed Student`s *t* test  *P* = 0.243 | NA | *Cyfip1*^WT^ n = 15,  *Cyfip1*^HET^ n = 15 |
| **Figure 2D**, Rotarod | *Cyfip1*^WT^  Trial 1; *W* = 0.934, *P* = 0.254  Trial 2; *W* = 0.950, *P* = 0.458  Trial 3; *W* = 0.983, *P* = 0.052  Trial 4; *W* = 0.853, *P* = 0.012  Trial 5; *W* = 0.906, *P* = 0.087  Trial 6; *W* = 0.905, *P* = 0.083  Trial 7; *W* = 0.922, *P* = 0.160  *Cyfip1*^HET^  Trial 1; *W* = 0.956, *P* = 0.628  Trial 2; *W* = 0.938, *P* = 0.359  Trial 3; *W* = 0.945, *P* = 0.445  Trial 4; *W* = 0.907, *P* = 0.124  Trial 5; *W* = 0.924, *P* = 0.219  Trial 6; *W* = 0.976, *P* = 0.938  Trial 7; *W* = 0.934, *P* = 0.312 | Mauchly`s Test of Sphericity  *Cyfip1*^WT^  *W* = 0.362, *χ^2^* = 14.018, *P* = 0.836  *Cyfip1*^HET^  *W* = 0.069, *χ^2^* = 31.521, *P* = 0.055 | Repeated measures ANOVA  Main effect of Trial, Pillai`s Trace  *Cyfip1*^WT^  *F* = 16.094, *P* = 0.000  Followed by Bonferroni corrected pairwise comparison  *Cyfip1*^HET^  *F* = 1.092, *P* = 0.434 | 1.000  0.247 | *Cyfip1*^WT^ n = 17,  *Cyfip1*^HET^ n = 15 |
| **Figure 2E**, Velocity | *Cyfip1*^WT^ : *W* = 0.939, *P* = 0.539  *Cyfip1*^HET^*:* *W* = 0.909, *P* = 0.098 | Levene`s test  *F* = 0.005, *P* = 0.947 | Two-tailed Student`s *t* test  *P* = 0.540 | NA | *Cyfip1*^WT^ n = ,  *Cyfip1*^HET^n = |
| **Figure 2F**, Latency to fall on trial 1 | *Cyfip1*^WT^ *W* = 0.934, *P* = 0.254  *Cyfip1*^HET^*W* = 0.956, *P* = 0.624 | Levene`s test  *F* = 0.706, *P* = 0.407 | Student`s t-test, two-tailed,  *P* = 0.169 | NA | *Cyfip1*^WT^ n = 17,  *Cyfip1*^HET^n = 15 |
| **Figure 3A M1** | *Cyfip1*^WT^ : *W* = 0.969, *P* = 0.645  *Cyfip1*^HET^: *W* = 0.957, *P* = 0.379 | Levene`s test  *F* = 0.028, *P* = 0.867 | Student`s *t*-test, two-tailed  *P* = 0.008 | NA | *Cyfip1*^WT^ : n = 24,  *Cyfip1*^HET^: n = 24 |
| **Figure 3A V1** | *Cyfip1*^WT^ : *W* = 0.980, *P* = 0.935  *Cyfip1*^HET^: *W* = 0.972, *P* = 0.755 | Levene`s test  *F* = 4.474, *P* = 0.041 | Mann-Whitney, two-tailed  *U* = 166.000, *P* = 0.174 | NA | *Cyfip1*^WT^ : n = 24,  *Cyfip1*^HET^: n = 24 |
| **Figure 3A CA1** | *Cyfip1*^WT^ : *W* = 0.984, *P* = 0.978  *Cyfip1*^HET^: *W* = 0.963, *P* = 0.604 | Levene`s test  *F* = 2.830, *P* = 0.101 | Student`s *t*-test, two-tailed  *P* = 0.365 | NA | *Cyfip1*^WT^ : n = 20,  *Cyfip1*^HET^: n = 20 |
| **Figure 3A CA3** | *Cyfip1*^WT^ : *W* = 0.975, *P* = 0.855  *Cyfip1*^HET^: *W* = 0.960, *P* = 0.545 | Levene`s test  *F* = 3.992, *P* = 0.053 | Student`s *t*-test, two-tailed  *P* = 0.067 | NA | *Cyfip1*^WT^ : n = 20,  *Cyfip1*^HET^: n = 20 |
| **Figure 3C** | Formation  *Cyfip1*^WT^ *W* = 0.814, *P* = 0.000  *Cyfip1*^HET^*W* = 0.731, *P* = 0.000  Elimination  *CYFIP1*^WT^ *W* = 0.877, *P* = 0.000  *Cyfip1*^HET^*W* = 0.894, *P* = 0.002 | Levene`s test  *F* = 2.762, *P* = 0.044 | Two-way ANOVA  Main effect of plasticity  *F* = 1.567, *P* = 0.213  Main effect genotype  *F* = 4.718, *P* = 0.031  Interaction plasticity x genotype  *F* = 0.020, *P* = 0.887 | 0.238  0.578  0.020 | *Cyfip1*^WT^ dendrites  n = 40,  *Cyfip1*^HET^dendrites n = 36 |
| **Figure 3 D** | Base line  *Cyfip1*^WT^ *W* = 0.814, *P* = 0.000  *Cyfip1*^HET^*W* = 0.731, *P* = 0.000  After training  *Cyfip1*^WT^ *W* = 0.761, *P* = 0.000  *Cyfip1*^HET^*W* = 0.866, *P* = 0.000 | Mauchly`s Test of Sphericity assumed | Repeated measures ANOVA  Main effect of spine formation  *F* = 25.737, *P* = 0.000  Interaction spine formation x genotype  *F* = 0.260, *P* = 0.612 | 0.999  0.079 | *Cyfip1*^WT^ dendrites  n = 40,  *Cyfip1*^HET^dendrites n = 36 |
| **Figure 4A** | Trial 1; *W* = 0.959, *P* = 0.809  Trial 2; *W* = 0.991, *P* = 0.994  Trial 3; *W* = 0.947, *P* = 0.629  Trial 4; *W* = 0.928, *P* = 0.537  Trial 5; *W* = 0.936, *P* = 0.605  Trial 6; *W* = 0.967, *P* = 0.873  Trial 7; *W* = 0.734, *P* = 0.009 | Mauchly`s Test of Sphericity  *W* = 0.000, *χ^2^* = 30.757, *P* = 0.132 | Repeated measures ANOVA  Within-Subject Effects  *F* = 15.261, *P* = 0.000 | 1.000 | *Cyfip1*^WT^ = 7 |
| **Figure 4B** | Trial 1; *W* = 0.934, *P* = 0.254  Trial 2; *W* = 0.950, *P* = 0.458  Trial 3; *W* = 0.983, *P* = 0.052  Trial 4; *W* = 0.853, *P* = 0.012  Trial 5; *W* = 0.906, *P* = 0.087  Trial 6; *W* = 0.905, *P* = 0.083  Trial 7; *W* = 0.922, *P* = 0.160 | *W* = 0.362, *χ^2^* = 14.018, *P* = 0.836 | Repeated measures ANOVA  Main effect of Trial, Pillai`s Trace  *Cyfip1*^WT^  *F* = 16.094, *P* = 0.000  Followed by Bonferroni corrected pairwise comparison | 1.000 | *Cyfip1*^WT^ n = 17, |
| **Figure 4C** | Trained *Cyfip1*^WT^ P60 see **4A**  Untrained *Cyfip1*^WT^ P60 see **2D** | Mauchly`s Test of Sphericity  *W* = 0.597 *χ^2^* = 10.199, *P* = 0.965 | Repeated measures ANOVA, Pillai`s Trace  Main effect of Trial,  *F* = 3.260, *P* = 0.025;  Interaction Trial x Training,  *F* = 2.650, *P* = 0.047  Followed by Bonferroni corrected pairwise comparison | 0.805  0.723 | Trained *Cyfip1*^WT^ n = 7,  Untrained *Cyfip1*^WT^ n = 17 |
| **Figure 4D** | Trained *Cyfip1*^HET^ P60 see **4B** Untrained *Cyfip1*^HET^ P60 see **2D** | Mauchly`s Test of Sphericity  *W* = 0.133, *χ^2^* = 33.893, *P* = 0.029; Greenhouse-Geisser ε = 0.641 | Repeated measures ANOVA  Within-Subject Effects, Greenhouse-Geisser test  Main effect of Trial  *F* = 6.269, *P* = 0.000  Interaction Trial x Training  *F* = 2.872, *P* = 0.030  Followed by Bonferroni corrected pairwise comparison | 0.982  0.741 | Trained *Cyfip1*^HET^ n = 6,  Untrained *Cyfip1*^HET^ n = 15 |
| **Supplementary Fig3**, Motor cortex | *Cyfip1*^WT^ : *W* = 0.928, *P* = 0.139  *Cyfip1*^HET^: *W* = 0.910, *P* = 0.064 | Levene`s test  *F* = 0.967, *P* = 0.332 | Student`s t-test, one-tailed  *F* = 0.967, *P* = 0.017 | NA | *Cyfip1*^WT^ : n=20,  *Cyfip1*^HET^: n=20 |
| **Supplementary Fig3**, Hippocampus | *Cyfip1*^WT^ : *W* = 0.895, *P* = 0.116  *Cyfip1*^HET^: *W* = 0.916, *P* = 0.254 | Levene`s test  *F* = 2.682, *P* = 0.115 | Student`s t-test, one-tailed  *F* = 2.682, *P* = 0.002 | NA | *Cyfip1*^WT^ : n=13,  *Cyfip1*^HET^: n=12 |
| **Supplementary Fig3**, Striatum | *Cyfip1*^WT^ : *W* = 0.499, *P* = 0.396  *Cyfip1*^HET^: *W* = 0.881, *P* = 0.040 | Levene`s test  *F* = 0.441, *P* = 0.512 | Mann-Whitney, one-tailed  *U* = 88.000, *P* = 0.069 | NA | *Cyfip1*^WT^ : n=16,  *Cyfip1*^HET^: n=16 |
| **Supplementary Fig3**, Thalamus | *Cyfip1*^WT^ : *W* = 0.836, *P* = 0.091  *Cyfip1*^HET^: *W* = 0.840, *P* = 0.099 | Levene`s test  *F* = 0.703, *P* = 0.418 | Student`s t-test, one-tailed  *F* = 0.703, *P* = 0.332 | NA | *Cyfip1*^WT^ : n=7,  *Cyfip1*^HET^: n=7 |
| **Supplementary Fig3**, Somato-sensory cortex | *Cyfip1*^WT^ : *W* = 0.809, *P* = 0.096  *Cyfip1*^HET^: *W* = 0.757, *P* = 0.007 | Levene`s test  *F* = 0.774, *P* = 0.396 | Mann-Whitney, one-tailed  *U* = 22.000, *P* = 0.500 | NA | *Cyfip1*^WT^ : n=5,  *Cyfip1*^HET^: n=9 |
| **Supplementary Fig3**, Cerebellum | *Cyfip1*^WT^ : *W* = 0.911, *P* = 0.189  *Cyfip1*^HET^: *W* = 0.812, *P* = 0.013 | Levene`s test  *F* = 0.000, *P* = 0.987 | Mann-Whitney, one-tailed  *U* = 58.000, *P* = 0.148 | NA | *Cyfip1*^WT^ : n=13,  *Cyfip1*^HET^: n=12 |
| **Supplementary Fig3**, Liver | *Cyfip1*^WT^ : *W* = 0.838, *P* = 0.030  *Cyfip1*^HET^: *W* = 0.972, *P* = 0.911 | Levene`s test  *F* = 1.447, *P* = 0.245 | Mann-Whitney, one-tailed  *U* = 48.500, *P* = 0.471 | NA | *Cyfip1*^WT^ : n=11,  *Cyfip1*^HET^: n=9 |
| **Supplementary Fig3**, Spleen | *Cyfip1*^WT^ : *W* = 0.905, *P* = 0.213  *Cyfip1*^HET^: *W* = 0.838, *P* = 0.055 | Levene`s test  *F* = 1.721, *P* = 0.206 | Student`s t-test, one-tailed  *F* = 1.721, *P* = 0.478 | NA | *Cyfip1*^WT^ : n=11,  *Cyfip1*^HET^: n=9 |
