# Supplementary material for: Behavioral training rescues motor deficits in Cyfip1 haploinsufficiency mouse model of autism spectrum disorders
Source: Transl Psychiatry. 2019 Jan 21;9:29. doi: 10.1038/s41398-018-0338-9 (PMC6341103; doi:10.1038/s41398-018-0338-9)
Supplement: Supplementary file 8 — Supplementary figures legend [file 41398_2018_338_MOESM8_ESM.docx]

**Supplementary Figure 1. A.** Western blot analysis showing that Cyfip1 is detected in the synaptosome fraction of striatum (St), cerebellum (Cb) and cortex (Cx) following immunoprecipitation with Neuroligin-3 (NL3) antibodies. Note that FMRP is not detected in co-immunoprecipitated proteins. **B.** Schematic representation of the ASD-associated proteins interacting with Neuroligin-3 in excitatory and inhibitory synapses of *Pvalb*-expressing cells, based on mass-spectrometry analyses (Supplementary Table 3) and western blot analyses.

**Supplementary Figure 2. A.** Western blot analysis showed that Neuroligin-3 (NL-3) is detected in VNO and cerebella of wild-type mice. Note that an unspecific band of higher molecular weight detected in VNO and cerebellum of mice lacking *Nlgn3* (*Nlgn3^y/-^*). **B.** Western blot analysis showed that PSD95 does not co-immunoprecipitate with Neuroligin-3 in the VNO. Note the presence of an unspecific band in VNO of *Nlgn3^y/-^* mice, that is not detected in the immunoprecipitation. **C.** Crossing mice expressing Cre recombinase in OMP-expressing neurons in the VNO fail to re-express Neuroligin-3. **D.** *In-situ* hybridization showed that *Nlgn3* mRNA is localized in the neuronal epithelium of VNO. Note that the absence of signal when using the sense probe.

**Supplementary Figure 3.** Western blot analysis of Cyfip1 protein levels in motor cortex (mCx), hippocampus (Hpc), striatum (Str), thalamus (Th), somatosensory cortex (sCx), cerebellum (Cb), liver (Liv) and spleen (Spl) of *Cyfip1*^WT^ (WT) and *Cyfip1*^Het^ (HET) mice.
